# Supplementary material for: Electron iso-density surfaces provide a thermodynamically consistent representation of atomic and molecular surfaces
Source: Nat Commun. 2024 Jul 19;15:6086. doi: 10.1038/s41467-024-50408-8 (PMC11271626; doi:10.1038/s41467-024-50408-8)
Supplement: Supplementary file 1 — Supplementary Information [file 41467_2024_50408_MOESM1_ESM.docx]

**Supplementary Information**

**Electron Iso-Density Surfaces Provide a Thermodynamically Consistent Representation of Atomic and Molecular Surfaces**

Amin Alibakhshi^1,2,3,4,*^, Lars V. Schäfer^1,*^

^1^Center for Theoretical Chemistry, Ruhr University Bochum, 44780 Bochum, Germany

^2^Lehrstuhl für Theoretische Chemie II, Ruhr University Bochum, 44780 Bochum, Germany

^3^Research Center Chemical Sciences and Sustainability, Research Alliance Ruhr, 44780 Bochum, Germany

^4^Research Center Trustworthy Data Science and Security, Technical University Dortmund, 44227 Dortmund, Germany

^*^Email: [amin.alibakhshi@ruhr-uni-bochum.de](mailto:amin.alibakhshi@ruhr-uni-bochum.de) , [lars.schaefer@ruhr-uni-bochum.de](mailto:lars.schaefer@ruhr-uni-bochum.de)

**Phase change data of N_2_, O_2_, and F_2_**

All phase change data are taken from the NIST website^[[1]](#footnote-1)^. A MATLAB script to calculate atomic radii based on these data is provided in next section.

| Table S1- Phase change data of homonuclear diatomic molecules N_2_, O_2_, and F_2_ used in estimation of vdW radii of N, O, and F elements | | |
| --- | --- | --- |
| Temperature (K) | Surface tension (N/m) | Vaporization enthalpy(J/mol) |
| N2 | | |
| 63.151 | 0.012205 | 6037.3 |
| 68.151 | 0.011011 | 5886.8 |
| 73.151 | 0.009842 | 5725.8 |
| 78.151 | 0.0087 | 5550.6 |
| 83.151 | 0.007586 | 5357.6 |
| 88.151 | 0.006504 | 5142.4 |
| 93.151 | 0.005457 | 4899.5 |
| 98.151 | 0.004448 | 4622.5 |
| 103.15 | 0.003483 | 4301.5 |
| 108.15 | 0.002568 | 3922.4 |
| 113.15 | 0.001714 | 3459.4 |
| 118.15 | 0.000938 | 2859.27 |
| 123.15 | 0.000279 | 1950.04 |
| O2 |  |  |
| 54.361 | 0.022601 | 7766.8 |
| 59.361 | 0.021227 | 7643.6 |
| 64.361 | 0.01987 | 7518.8 |
| 69.361 | 0.01853 | 7392.3 |
| 74.361 | 0.017207 | 7263.7 |
| 79.361 | 0.015902 | 7131 |
| 84.361 | 0.014617 | 6991.7 |
| 89.361 | 0.013353 | 6843.2 |
| 94.361 | 0.01211 | 6682.5 |
| 99.361 | 0.01089 | 6506.7 |
| 104.36 | 0.009695 | 6313.4 |
| 109.36 | 0.008526 | 6099 |
| 114.36 | 0.007386 | 5860.5 |
| 119.36 | 0.006277 | 5593.3 |
| 124.36 | 0.005204 | 5291.7 |
| 129.36 | 0.00417 | 4947.5 |
| 134.36 | 0.003181 | 4548.3 |
| 139.36 | 0.002246 | 4073.2 |
| 144.36 | 0.001379 | 3481.34 |
| 149.36 | 0.000605 | 2668.82 |
| 154.36 | 1.25E-05 | 810.55 |
| F2 |  |  |
| 53.481 | 0.022646 | 7564 |
| 58.481 | 0.021138 | 7429.4 |
| 63.481 | 0.01965 | 7292.8 |
| 68.481 | 0.018181 | 7151.09 |
| 73.481 | 0.016734 | 7003.31 |
| 78.481 | 0.015308 | 6847.13 |
| 83.481 | 0.013906 | 6680.008 |
| 88.481 | 0.012529 | 6499.31 |
| 93.481 | 0.011179 | 6302.53 |
| 98.481 | 0.009857 | 6087.17 |
| 103.48 | 0.008566 | 5850.3 |
| 108.48 | 0.007309 | 5588.1 |
| 113.48 | 0.00609 | 5295 |
| 118.48 | 0.004913 | 4963.2 |
| 123.48 | 0.003785 | 4579.7 |
| 128.48 | 0.002714 | 4123.4 |
| 133.48 | 0.001716 | 3554.6 |
| 138.48 | 0.000815 | 2778.9 |
| 143.48 | 8.56E-05 | 1290.8 |
|  |  |  |

**MATLAB script to calculate atomic radii from the phase change data for N**

For a diatomic molecule, calculation of the total surface from inter-atomic distance and vdW radii of the atoms follows simple geometrical calculations (calculation of spherical cap surface). A MATLAB script which calculates vdW surfaces for a range of vdW radii and takes the one for which the calculated vdW surface is closest to the TE surface is provided in the following:

%%%%%%%%%%%%%%%%%%%%%%%%%%%%%%%%%%%%%%%%%%%%%%%%%%%%%%%%%%%%%%%%%%%%%

clear

clc

format short G

d=load(N2.txt'); % columns are T, St, and H_vap

%%%%%%%%%%%%%%%%%%%%%%%%%%%%%%%%%%%%%%%%%%%%%%%%%%%%%%%%%%%%%%%%%%%%%

d_bond=1.4119; %bond length from NIST database

tc=144; % critical temperature from NIST database

%%%%%%%%%%%%%%%%%%%%%%%%%%%%%%%%%%%%%%%%%%%%%%%%%%%%%%%%%%%%%%%%%%%%%

t=d(:,1);

st=d(:,2);

gama0=((1-t./tc).^(11/9))\st;

hv=d(:,3);

S_TE=(0.5*gama0*(2*(1-t/tc).^(11/9)+11/9*t.*(1t/tc).^(2/9)./tc))\(hv+8.314/2.*t.*log(t/tc));

S_TE=S_TE/6.022e23*1e20;

out=NaN(8005,2);

ii=1;

for r_atom=1.3:0.0001:2.1

r1=r_atom;

r2=r_atom;

cos_alpha1=(r2^2-r1^2-d_bond^2)/(-2*r1*d_bond);

cos_alpha2=(r1^2-r2^2-d_bond^2)/(-2*r2*d_bond);

s_vdw=4*pi*(r1^2+r2^2)-2*pi*r1^2*(1-cos_alpha1)-2*pi*r2^2*(1-cos_alpha2);

out(ii,1)=r_atom;

out(ii,2)=abs(s_vdw-S_TE);

ii=ii+1;

end

out=out(1:ii-1,:);

f=find(out(:,2)==min(out(:,2)));

out(f,:);

r_element=out(f,1)

%%%%%%%%%%%%%%%%%%%%%%%%%%%%%%%%%%%%%%%%%%%%%%%%%%%%%%%%%%%%%%%%%%%%%

**Orca and Multiwfn scripts to calculate iso-density surfaces**

A sample script used by us to compute .gbw files by Orca is provided in the following.

###################################################################

! dsd-pbep86 def2-qzvpd def2-tzvpd/c d3bj nofrozencore PModel verytightscf PAL16

%maxcore 1500

%mp2 Density relaxed end

%scf

guessmode Cmatrix

STABPerform true

STABRestartUHFifUnstable true

End

*xyzfile molecule.xyz # xyz file of the molecule

###################################################################

Afterwards, the generated .gbw can be converted to .wfn file via orca_2aim tool which then can be processed by Multiwfn to compute total iso-density surface via the following Bash command:

$Multiwfn_path/Multiwfn xxx.wfn << EOF > out.log

12

1

1

0.0025

3

0.1

0

-1

-1

q

EOF

**Computed TE and iso-density surfaces for the benchmark set**

| Table S2. Iso-density and TE surfaces evaluated for the 104 studied molecules. For iso-density surfaces, a cut-off density of 0.0016 (a.u.) is used and surfaces are given in Å^2^. | | | | | |
| --- | --- | --- | --- | --- | --- |
|  | **experiment (TE)** | **iso- density** |  | **experiment (TE)** | **iso- density** |
| 1,1-DIMETHOXYETHANE | 131.67 | 133.39 | BUTANE | 113.80 | 115.47 |
| 1,1-DIMETHYLCYCLOHEXANE | 159.11 | 162.83 | BUTANETHIOL | 137.50 | 137.67 |
| 1,1-DIMETHYLCYCLOPENTANE | 148.10 | 150.58 | BUTYL ACETATE | 175.57 | 167.49 |
| 1,2,4-TRIMETHYLBENZENE | 177.83 | 170.67 | cis-1,2-DIMETHYLCYCLOHEXANE | 160.01 | 163.25 |
| 1,2-DIMETHYLBENZENE | 155.78 | 151.11 | cis-1,3-DIMETHYLCYCLOHEXANE | 168.09 | 168.45 |
| 1,3-CYCLOHEXADIENE | 116.08 | 122.70 | cis-1,4-DIMETHYLCYCLOHEXANE | 163.06 | 165.81 |
| 1,4-DIOXANE | 118.96 | 117.16 | cis-2-BUTENE | 108.37 | 108.26 |
| 1-BUTENE | 104.38 | 108.42 | CYCLOHEPTANE | 147.05 | 147.65 |
| 1-HEPTENE | 162.13 | 165.57 | CYCLOHEXANE | 126.47 | 132.90 |
| 1-HEXANETHIOL | 174.79 | 175.12 | CYCLOHEXANONE | 143.25 | 136.63 |
| 1-OCTENE | 180.69 | 185.87 | CYCLOHEXYLAMINE | 141.23 | 145.40 |
| 1-PENTANETHIOL | 156.51 | 157.20 | DIBORANE | 81.38 | 81.94 |
| 1-PROPYNE | 86.04 | 82.52 | DIETHYL DISULFIDE | 158.82 | 158.49 |
| 2,2,3,3-TETRAMETHYLPENTANE | 184.24 | 182.26 | DIETHYL ETHER | 128.39 | 128.28 |
| 2,2,3-TRIMETHYLPENTANE | 176.80 | 174.78 | DIETHYL SULFIDE | 136.27 | 137.86 |
| 2,2,4,4-TETRAMETHYLPENTANE | 188.03 | 188.26 | ETHANE | 75.09 | 75.61 |
| 2,2,4-TRIMETHYLPENTANE | 179.23 | 178.82 | ETHANETHIOL | 100.98 | 99.65 |
| 2,2,5-TRIMETHYLHEXANE | 201.76 | 199.50 | ETHYL PROPIONATE | 152.67 | 148.61 |
| 2,2-DIMETHYLBUTANE | 147.41 | 144.76 | ETHYLCYCLOHEXANE | 163.02 | 168.06 |
| 2,2-DIMETHYLHEXANE | 185.38 | 183.84 | ETHYLCYCLOPENTANE | 152.88 | 154.17 |
| 2,2-DIMETHYLPENTANE | 165.54 | 164.44 | ETHYLENE | 67.47 | 67.87 |
| 2,3,3-TRIMETHYLPENTANE | 173.49 | 172.17 | FURAN | 97.71 | 95.77 |
| 2,3,4-TRIMETHYLPENTANE | 176.71 | 176.67 | HEPTANE | 169.07 | 172.67 |
| 2,3-DIMETHYLBUTANE | 146.33 | 145.67 | HEXANE | 149.03 | 153.65 |
| 2,3-DIMETHYLHEXANE | 181.22 | 181.90 | HYDROGEN SULFIDE | 58.66 | 57.85 |
| 2,3-DIMETHYLPENTANE | 161.37 | 162.83 | ISOBUTANE | 112.75 | 113.68 |
| 2,4-DIMETHYLHEXANE | 181.69 | 184.43 | ISOBUTENE | 107.51 | 108.04 |
| 2,4-DIMETHYLPENTANE | 166.81 | 166.84 | ISOPENTANE | 129.36 | 131.21 |
| 2,5-DIMETHYLHEXANE | 186.74 | 185.38 | ISOPROPYLBENZENE | 171.65 | 170.14 |
| 2,6-DIMETHYLHEPTANE | 206.25 | 203.98 | ISOPROPYLCYCLOPENTANE | 164.45 | 168.54 |
| 2-METHYL THIOPHENE | 130.39 | 127.61 | METHYL n-BUTYRATE | 151.81 | 147.64 |
| 2-METHYL-2-BUTENE | 126.14 | 126.87 | METHYL PROPIONATE | 134.06 | 128.31 |
| 2-METHYLHEPTANE | 188.43 | 188.72 | METHYLCYCLOHEXANE | 145.75 | 150.51 |
| 2-METHYLHEXANE | 166.39 | 169.16 | METHYLCYCLOPENTANE | 131.09 | 136.27 |
| 2-METHYLOCTANE | 207.01 | 207.92 | n-PROPYLMERCAPTAN | 119.67 | 118.41 |
| 2-METHYLPENTANE | 149.60 | 150.22 | o-ETHYLTOLUENE | 171.52 | 168.92 |
| 2-METHYL-PROPANETHIOL | 141.32 | 134.30 | PENTANE | 132.99 | 134.99 |
| 2-METHYLTHIOLANE | 142.24 | 138.48 | PENTYLAMINE | 152.56 | 148.00 |
| 3,3-DIETHYLPENTANE | 188.01 | 188.72 | PHENYLTHIOL | 140.18 | 138.31 |
| 3,3-DIMETHYLHEXANE | 181.10 | 179.34 | PROP-1-ENE | 87.61 | 89.03 |
| 3,3-DIMETHYLPENTANE | 157.31 | 159.74 | PROPANE | 94.75 | 95.87 |
| 3,4-DIMETHYLHEXANE | 178.79 | 179.52 | PROPANE-2-THIOL | 120.49 | 117.21 |
| 3-ETHYL-2-METHYLPENTANE | 178.68 | 179.34 | PROPYL ACETATE | 155.78 | 148.33 |
| 3-ETHYL-3-METHYLPENTANE | 174.37 | 174.38 | PROPYLBENZENE | 171.78 | 172.07 |
| 3-ETHYLHEXANE | 182.36 | 184.78 | PROPYLCYCLOPENTANE | 171.75 | 173.40 |
| 3-ETHYLPENTANE | 160.48 | 165.28 | PYRIDINE | 113.21 | 110.97 |
| 3-METHYL-1-BUTENE | 127.79 | 126.61 | TETRAHYDROFURAN | 110.54 | 110.00 |
| 3-METHYLHEPTANE | 186.74 | 186.86 | THIACYCLOPENTANE | 117.08 | 120.77 |
| 3-METHYLHEXANE | 166.89 | 167.65 | THIOPHENE | 109.54 | 107.24 |
| 3-METHYLPENTANE | 148.02 | 148.63 | trans-1,2-DIMETHYLCYCLOHEXANE | 163.44 | 165.72 |
| 3-METHYLTHIOPHENE | 129.49 | 127.71 | trans-1,3-DIMETHYLCYCLOHEXANE | 161.85 | 165.69 |
| 4-METHYLHEPTANE | 187.76 | 187.59 | trans-1,4-DIMETHYLCYCLOHEXANE | 167.88 | 168.47 |
|  |  |  |  |  |  |
|  |  |  |  |  |  |

| Table S3. Iso-density and TE surfaces evaluated for the data set containing 184 molecules. For iso-density surfaces, a cut-off density of 0.0016 (a.u.) is used and surfaces are given in Å^2^. | | | | | |
| --- | --- | --- | --- | --- | --- |
|  | **experiment (TE)** | **iso- density** |  | **experiment (TE)** | **iso- density** |
| 1,1,1,2,2-PENTAFLUOROPROPANE | 98.98 | 117.4878 | CHLOROBENZENE | 132.8 | 133.1658 |
| 1,1,1,2,3,3,3-HEPTAFLUOROPROPANE | 127.08 | 126.466 | CHLORODIFLUOROMETHANE | 88.563 | 83.60306 |
| 1,1,1,2,3,3-HEXAFLUOROPROPANE | 129.76 | 122.7596 | CHLOROFORM | 109.31 | 109.1192 |
| 1,1,1,2-TETRAFLUOROETHANE | 104.77 | 95.46602 | CHLOROPENTAFLUOROETHANE | 115.12 | 116.1592 |
| 1,1,1-TRICHLOROETHANE | 120.33 | 125.4655 | CHLOROTRIFLUOROMETHANE | 83.74 | 88.08062 |
| 1,1,1-TRIFLUOROETHANE | 97.876 | 89.98466 | CHLOROTRIMETHYLSILANE | 146.18 | 148.4942 |
| 1,1,2,2-TETRAFLUOROETHANE | 92.271 | 95.85999 | cis-1,2-DICHLOROETHYLENE | 106.7 | 103.8768 |
| 1,1,2-TRICHLOROTRIFLUOROETHANE | 134.58 | 137.1934 | cis-2-PENTENE | 123.14 | 128.6771 |
| 1,1-DICHLORO-1,2,2,2-TETRAFLUOROETHANE | 127.65 | 126.8311 | cis-DECAHYDRONAPHTHALENE | 178.29 | 181.6869 |
| 1,1-DICHLORO-1-FLUOROETHANE | 115.42 | 114.6918 | DECANE | 228.7 | 234.2908 |
| 1,1-DICHLORO-2,2,2-TRIFLUOROETHANE | 126.41 | 123.0359 | DIETHYL KETONE | 152.69 | 136.8029 |
| 1,1-DICHLOROETHANE | 109.19 | 110.6846 | DIMETHYL FORMAMIDE | 140.02 | 111.2424 |
| 1,1-DIETHOXYETHANE | 173.34 | 174.4602 | ETHYL CHLORIDE | 94.294 | 94.0208 |
| 1,1-DIFLUOROETHANE | 102.08 | 85.57947 | ETHYL FORMATE | 118.25 | 108.4441 |
| 1,2,3,4,5,6-HEXAFLUOROBENZENE | 142.81 | 145.4734 | ETHYL ISOVALERATE | 182.9 | 184.4244 |
| 1,2,3,4-TETRAHYDRONAPHTHALENE | 184.59 | 172.3758 | ETHYL n-BUTYRATE | 172.37 | 167.8635 |
| 1,2,3-TRIMETHYLBENZENE | 175.2 | 167.4504 | ETHYL VINYL ETHER | 113.72 | 118.9331 |
| 1,2,4,5-TETRAMETHYLBENZENE | 196.48 | 186.7221 | ETHYLACETYLENE | 107.06 | 103.5406 |
| 1,3-DIMETHYLADAMANTANE | 199.92 | 198.3056 | ETHYLAMINE | 102 | 90.45542 |
| 1,3-DIMETHYLBENZENE | 157.57 | 154.5617 | ETHYLBENZENE | 153.02 | 154.0031 |
| 1,4-DIMETHYLBENZENE | 159.98 | 154.6485 | ETHYL-tert-PENTYL ETHER | 153.44 | 175.144 |
| 1,5-HEXADIENE | 134.99 | 141.4898 | FLUOROBENZENE | 123.26 | 120.4752 |
| 1-BROMOBENZENE | 136.51 | 138.9497 | FURFURAL | 125.12 | 119.6253 |
| 1-BROMOPROPANE | 121.32 | 118.8274 | HEXANENITRILE | 185.11 | 155.6387 |
| 1-CHLORO-1,1-DIFLUOROETHANE | 106.9 | 102.8892 | HYDRAZINE | 73.963 | 64.06602 |
| 1-CHLOROPENTANE | 150.39 | 151.7122 | INDAN | 168.44 | 158.4599 |
| 1-DECENE | 215.42 | 225.1792 | ISOBUTYL ACETATE | 172.79 | 165.2588 |
| 1-METHOXY BUTANE | 143.38 | 146.408 | ISOBUTYL FORMATE | 160.51 | 144.9322 |
| 1-METHOXY PENTANE | 158 | 165.3363 | ISOBUTYLBENZENE | 191.37 | 186.6912 |
| 1-METHOXY PROPANE | 122.77 | 126.8823 | ISOPROPYL ACETATE | 152.16 | 147.5958 |
| 1-METHYL-2-PYRROLIDINONE | 157.93 | 136.0517 | ISOPROPYLAMINE | 136.48 | 108.7011 |
| 1-NONENE | 195.47 | 205.81 | m-CRESOL | 183.21 | 144.0719 |
| 1-PENTENE | 118.34 | 128.0708 | m-DICHLOROBENZENE | 142.11 | 150.8487 |
| 2- METHYLPROPYL 2-METHYLPROPANOATE | 208.18 | 201.7302 | MESITYLENE | 181.06 | 174.0119 |
| 2,2,3,4-TETRAMETHYLPENTANE | 186.39 | 187.1275 | METHYL ACETATE | 116.41 | 108.5861 |
| 2,2,3-TRIMETHYLBUTANE | 153.46 | 157.3433 | METHYL CHLORIDE | 79.988 | 73.36805 |
| 2,2-DIMETHYLHEPTANE | 200.14 | 203.0568 | METHYL ETHYL ETHER | 105.82 | 107.3822 |
| 2,3,3,4-TETRAMETHYLPENTANE | 184.87 | 184.6428 | METHYL ETHYL SULFIDE | 117.82 | 118.8278 |
| 2,3-XYLENOL | 212.91 | 158.4452 | METHYL FORMATE | 94.52 | 87.61783 |
| 2,4-XYLENOL | 212.68 | 162.1347 | METHYL ISOBUTYRATE | 147.14 | 146.1326 |
| 2,5-XYLENOL | 186.56 | 161.9452 | METHYL-(2-PROPYL) ETHER | 128.5 | 124.319 |
| 2,6-DIMETHYLPYRIDINE | 145.2 | 150.9387 | METHYLAMINE | 81.452 | 69.85015 |
| 2,7-DIMETHYLNAPHTHALENE | 215.02 | 199.5776 | METHYLAMINOBENZENE | 158.35 | 148.2664 |
| 2-BUTANONE | 133.1 | 117.5837 | METHYLENEDIOXYDIMETHANE | 118.36 | 115.8607 |
| 2-BUTOXYETHYLACETATE | 197.85 | 213.8428 | METHYLISOPROPYL KETONE | 146.7 | 134.2396 |
| 2-CHLORO-1,1,1,2-TETRAFLUOROETHANE | 116.68 | 112.0607 | NITROSYL CHLORIDE | 70.667 | 72.11973 |
| 2-ETHOXY-2-METHYLPROPANE | 142.68 | 159.2667 | o-TOLUIDINE | 160.03 | 146.1999 |
| 2-ETHOXYETHYL ACETATE | 189.9 | 179.1743 | OXIRANE | 83.516 | 74.58548 |
| 2-ETHYLPHENOL | 184.44 | 160.5474 | p-CRESOL | 184.81 | 144.1352 |
| 2-HEPTANONE | 186.63 | 174.455 | p-CYMENE | 188.4 | 189.8135 |
| 2-HEXANONE | 168.54 | 154.9351 | p-DICHLOROBENZENE | 151.71 | 150.8861 |
| 2-METHOXY-2-METHYLPROPANE | 127.86 | 138.3806 | p-DIETHYLBENZENE | 197.83 | 192.5602 |
| 2-METHOXYETHAN-1-OL | 150.75 | 116.0259 | PENTANENITRILE | 164.81 | 136.4667 |
| 2-METHYL-1-BUTENE | 122.89 | 126.0923 | PERCHLORYL FLUORIDE | 79.19 | 83.37901 |
| 2-METHYL-2-BUTANOL | 188.1 | 137.1228 | p-ETHYLTOLUENE | 178.21 | 173.5915 |
| 2-METHYLOXIRAN | 100.02 | 95.55331 | PHENANTHRENE | 211.05 | 203.0407 |
| 2-METHYLOXOLANE | 122.95 | 128.9025 | PHENETOLE | 167.87 | 165.3456 |
| 2-METHYLPYRIDINE | 135.19 | 131.0476 | PHENOL | 151.57 | 124.537 |
| 2-NONANONE | 225.2 | 214.8548 | PHOSGENE | 94.362 | 94.10094 |
| 2-PENTANONE | 155.56 | 136.6827 | PHOSPHINE | 62.127 | 64.57436 |
| 3,5-XYLENOL | 217.45 | 163.5473 | PIPERIDINE | 131.07 | 128.6912 |
| 3-ETHYLPHENOL | 208.87 | 162.8199 | PROPANAL | 106.63 | 98.23709 |
| 3-HEXANONE | 166.78 | 155.2909 | PROPIONITRILE | 129.49 | 97.80076 |
| 3-METHYLPYRIDINE | 137.83 | 130.7311 | PROPYL CHLORIDE | 113.67 | 113.1677 |
| 4-METHYL-2-PENTANOL | 216.41 | 158.311 | PROPYL FORMATE | 139.97 | 127.3473 |
| 4-METHYLPYRIDINE | 139.42 | 130.7361 | PROPYL n-BUTYRATE | 181.52 | 186.3597 |
| 5-NONANONE | 222.56 | 212.1313 | PROPYL PROPIONATE | 169.81 | 167.4999 |
| ACETALDEHYDE | 91.8 | 78.45931 | PROPYLAMINE | 118.41 | 109.8284 |
| ACETONE | 118.35 | 98.38755 | PROPYLENE GLYCOL MONOMETHYL ETHER ACETATE | 182.36 | 176.791 |
| ACETOPHENONE | 159.36 | 154.3505 | p-TOLUIDINE | 165.42 | 148.831 |
| ALLENE | 92.178 | 82.53528 | PYRROLE | 125.48 | 99.89889 |
| AMMONIA | 57.803 | 46.55153 | PYRROLIDINE | 118.62 | 113.8351 |
| ANILINE | 139.54 | 129.2303 | QUINOLINE | 166.28 | 156.6681 |
| ANISOLE | 144.16 | 144.3813 | sec-BUTYL CHLORIDE | 129.97 | 129.8324 |
| ARSINE | 65.427 | 69.04199 | sec-BUTYLAMINE | 134.35 | 126.6141 |
| BENZALDEHYDE | 145.61 | 137.0704 | sec-BUTYLBENZENE | 186.66 | 186.9633 |
| BENZENE | 114.68 | 115.4989 | tert-BUTYLAMINE | 138.89 | 124.5994 |
| BENZOPHENONE | 215.65 | 211.2421 | tert-BUTYLBENZENE | 185.76 | 182.3211 |
| BIPHENYL | 190.85 | 191.5025 | TETRAETHYLSILANE | 202.58 | 215.6798 |
| BORON TRICHLORIDE | 105.43 | 107.6413 | TETRAMETHYLSILANE | 141.81 | 152.2436 |
| BROMODIFLUOROMETHANE | 91.693 | 89.53295 | THIONYL CHLORIDE | 90.795 | 104.2471 |
| BROMOETHANE | 104.15 | 99.89827 | TOLUENE | 135.51 | 135.0543 |
| BROMOTRIFLUOROMETHANE | 87.984 | 93.78555 | trans-1,2-DICHLOROETHYLENE | 92.238 | 105.6714 |
| BUTANAL | 119.4 | 117.7445 | trans-2-PENTENE | 129.13 | 129.5935 |
| BUTANE, 2-METHOXY-2-METHYL | 138.43 | 154.2439 | trans-DECAHYDRONAPHTHALENE | 184.72 | 185.3903 |
| BUTANENITRILE | 144.72 | 117.0401 | TRICHLOROFLUOROMETHANE | 107.48 | 113.0892 |
| BUTYL CHLORIDE | 131.35 | 132.6723 | TRIDECANE | 286.17 | 275.0986 |
| BUTYL PROPIONATE | 190.45 | 186.8424 | TRIETHYLAMINE | 154.68 | 162.8843 |
| BUTYLAMINE | 137.21 | 128.9558 | TRIFLUOROMETHANE | 79.903 | 69.29114 |
| BUTYLBENZENE | 193.17 | 191.0238 | TRIMETHYLAMINE | 112.15 | 108.7278 |
| CARBON DISULFIDE | 81.278 | 89.11648 | TRIPROPYLAMINE | 203.09 | 215.594 |
| CARBON TETRACHLORIDE | 117.88 | 124.0585 | UNDECANE | 249.44 | 253.852 |
|  |  |  |  |  |  |
|  |  |  |  |  |  |

1. <https://webbook.nist.gov/chemistry/fluid/>). [↑](#footnote-ref-1)
