# Supplementary material for: Electron iso-density surfaces provide a thermodynamically consistent representation of atomic and molecular surfaces
Source: Nat Commun. 2024 Jul 19;15:6086. doi: 10.1038/s41467-024-50408-8 (PMC11271626; doi:10.1038/s41467-024-50408-8)
Supplement: Supplementary file 3 — Description of Additional Supplementary Files [file 41467_2024_50408_MOESM3_ESM.pdf]

File Name: Supplementary Data 1.xlsx
Description: Computed iso-density and experimentally determined surfaces for each studied molecule

File Name: Supplementary Data 2
Description: xyz coordinates of all studied molecules

File Name: Source Data.xlsx
Description: Source data used in figures 1 and 2

File Name: Supplementary Software
Description: Scripts and sample input file for running Multiwfn code (Multiwfn.in), Orca code (orca_input.in), and Matlab code for computing molecular surfaces (analyze_surf.m)
